# Emergence of informative higher scales in biological systems: a computational toolkit for optimal prediction and control

**DOI:** 10.1080/19420889.2020.1802914

**Published:** 2020-08-15

**Authors:** Erik Hoel, Michael Levin

**Affiliations:** a Allen Discovery Center, Tufts University, Medford, MA, USA; b Wyss Institute for Biologically Inspired Engineering, Harvard University, Boston, MA, USA

**Keywords:** Emergence, complexity, information, quantitative, causation, network

## Abstract

The biological sciences span many spatial and temporal scales in attempts to understand the function and evolution of complex systems-level processes, such as embryogenesis. It is generally assumed that the most effective description of these processes is in terms of molecular interactions. However, recent developments in information theory and causal analysis now allow for the quantitative resolution of this question. In some cases, macro-scale models can minimize noise and increase the amount of information an experimenter or modeler has about “what does what.” This result has numerous implications for evolution, pattern regulation, and biomedical strategies. Here, we provide an introduction to these quantitative techniques, and use them to show how informative macro-scales are common across biology. Our goal is to give biologists the tools to identify the maximally-informative scale at which to model, experiment on, predict, control, and understand complex biological systems.

## Introduction

A “big data” approach has become standard in the biological sciences over the past decade [1,2]. As techniques improve, ever more emphasis is placed on understanding, in the most fine-grained possible manner, the molecular and genetic pathways of life [3,4]. Yet, such an approach often leads to a bewildering complexity as models of biological systems grow to a significant dimensionality. This poses particular problems for asking “what does what” in terms of cellular mechanisms, regulation, or development. How should modelers and experimenters proceed to build the best possible models of such systems and pathways, particularly when what’s necessary for understanding are causal models like interactomes, from which we hope to derive actionable policies for prediction and control in biomedical settings?

Here we focus on models that describe the relationships within a biological system, particularly models used for understanding “what does what.” We refer to these as *causal* models. A biological system’s causal model can be revealed through interventions and observing their effects. This can be done via the up- or down-regulation of genes [5], optogenetic stimulation of neurons [6], transcranial magnetic stimulation [7], a randomized drug trial [8], a modulation of endogenous bioelectric networks [9,10], or a genetic knockout or knock-in [11], among many other techniques common across the biological sciences. In general, to establish a causal model, an interventional approach is needed [12]. Such a causal model might be a gene regulatory network [13] or protein interactomes [14]. In general, biological causal models, such as Bayesian networks, can be reconstructed from intervention, observation, and time-series data [15].

However, it may be the case that the most complex, that is, the most fine-grained and detailed causal model possible, can actually harm understanding of what does what in some biological system. One reason is that extremely complex and fine-grained models may contain within them an overwhelming amount of noise. Note that we do not here mean “noise” in that the model is not an accurate description of reality. Instead, fine-grained models, even if highly accurate, will often have intrinsic noise in the form of uncertainty in state-transitions (such as in a gene regulatory network, GRN, wherein many genes might be upregulated probabilistically) or uncertainty in binding (such as in a protein interactome wherein one protein may bind to many others and it cannot be known ahead of time which it will bind to). This means there can be uncertainty of the effect of an experimenter’s interventions on any part of the model. Sources of randomness include how cell molecules are subjected to Brownian motion [16], the stochastic nature of ion channels [17], and chaotic dynamics such as in the brain [18]. Many biological systems also possess significant degeneracy, from genes to neural networks [19]. Degeneracy is also a form of uncertainty or noise in that, given a particular output, it could have come from many different inputs. Ultimately, as open systems, the intrinsic interactions of organisms and cells are always exposed to the noise of the world and so become noisy themselves. This amount of intrinsic noise in biology, and therefore uncertainty, can be understood to be the central problem for modeling and understanding “what does what” in biological systems. We are concerned with this question: How can complex models be analyzed and built in a way that minimizes noise?

A key insight for solving this issue is that many systems have multiple levels of valid description and interpretation, that is, they have different *scales*. A computer can be described at the scale of its wiring, its machine code, or its user interface. An organism can be described at the scale of its underlying chemistry, its genotype, or its physiological or anatomical phenotype. Which of these descriptions provide the best understanding of “what does what”? The answer to this question requires a formal way of modeling the given system at different scales, such as a micro- or macro-scale. A *micro-scale* is some “lower level” of a system wherein it is modeled in the most fine-grained and detailed manner possible. A *macro-scale* is some coarse-grained or dimensionally-reduced “higher level” model of the system.

Different models at different scales are common across biology. For instance, neuroscience has long accommodated work that spans across multiple scales. Research at the micro-scale of the brain includes everything from examining molecular networks of cytoskeletal signaling to neurotransmitter receptor proteins in neurons. Indeed, in the brain there is a rich repertoire of individual variation yet global functions remains highly similar [20]. For instance, neurons may perform a set of individual computations while the larger circuit they are part of performs an entirely different computation at the higher level [21]. In fact, rat cortical neurons left to develop spontaneously *in vivo* migrate to form a clear macro-scale architecture of connectivity, indicating that the advantages of multi-scale structure might be built into developmental preferences [22]. And even brain imaging devices themselves span a significant spatiotemporal range, which necessarily leads to differences in models of functional connectivity in the brain [23]. Without a clear best spatiotemporal scale for understanding brain function, the debate rages on as to whether all of the higher level system functions are ultimately best expressed as molecular dynamics or at the level of individual neurons [24–27].

A rich literature exists regarding levels of explanation in biology, and whether molecular explanations are always to be preferred [28–36]. Some have argued that such reduction is not always a universally optimal strategy, particularly in biology due to the adaptive self-organizing nature of organism and cellular development, function, and behavior [37–40]. While the great majority of the community has settled on the level of molecules as the gold standard for biological models, it is becoming clear that even when all molecular-level fine-grained details and pathways are known, biology does not always carve neatly at any obvious joints. For example, it now seems very possible that there is no underlying shared molecular cellular identity (e.g. as being compiled by “cell-atlas” studies [41]. Generally, a preference for micro-scale models in biology is often just an expression of the assumption that the best possible model of any physical system, at least in principle, is at a level as fine-grained and detailed as possible [42,43].

Until recently, the question of which level of explanation is “best” has been a philosophical one, debated based on *a priori* preferences in different fields. However, recent advances in information theory now make it possible to provide a rigorous, objective analysis for identifying the most informative causal model governing a given phenomenon. This can allow for identifying the best scale for experimental inverventions, prediction and retrodiction, or asking “what does what” within a model, which are central questions for scientific models and understanding. Here, we provide an introduction and a primer for the use of these new techniques, and show how to identify situations where there are informative higher scales available for causal modeling and experimental intervention. Specifically, we offer tools to identify when predictive, efficient, and informative higher scales emerge from lower ones. We argue that identifying optimal informative models of biological systems should be a standard tool of analysis for experimenters and modelers dealing with complex multi-level systems. This approach is based on the fact that macro-scales can be modeled explicitly as coarse-grains, averages, or dimensional reductions.

## Defining macro-scale and micro-scale biological models

**Figure 1.**
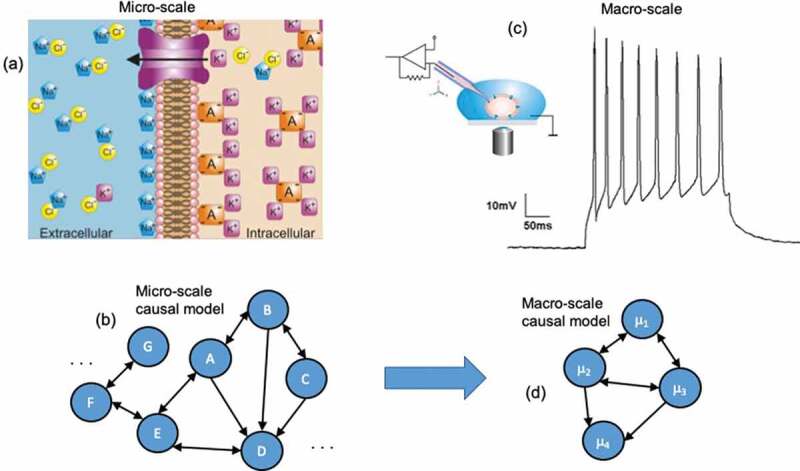
Comparing micro to macro. (a) A biological micro-scale, here a set of ion channels opening and closing, which makes up the membrane potential. (b) A causal model, and abstraction of the workings of the system at the micro-scale, is created by the modeler or experimenter (generally via interventions). This causal model might represent the openings and closings of channels, or the interactions of other molecular interactions, and may have a very high number of parameters. (c) Biological systems often have available macro-scales which are some dimension reduction of the micro-scale. An example might be the membrane potential of a cell. Often these biological macro-scales have interventions that manipulate them directly, such as current injection to change the variables or states can only be manipulated at the macro-scale. (d) A macro-scale causal model is an abstraction, wherein each variable or element might represent that state of the macro-scale and the effects of changes of those states, like how increases the membrane potential might lead to further changes in neighboring cells.

In a sense, all models used in biology are macro-scales, since they are not physical models. No experimenter or theorist would consider modeling a cell at the scale of quarks. Therefore, terms like “micro” and “macro” are fundamentally relative to one another in biology. The terms refer to different descriptions of the same system at different levels of detail: a micro-scale might be the full set of all molecular interactions and ion channels opening and closing within a network of cells, whereas a macro-scale might be some coarse-grain or dimension reduction of the same set of cells, such as the dynamics of their membrane potentials. In general, macro-scales are multiply-realizable: many different combinations of ion channel openings might lead to the same membrane potential. It is possible that macro-states such as resting potential [44] or pressure [45] can serve as convenient and tractable control points of decision-making by cells and tissues [46] as opposed to molecular pathways. For instance, manipulating the bioelectric field of a developing tissue ignores the underlying ion channel changes [9,10]. In such a case, the micro-scale in a set of cells would be the underlying ion channel changes (Figure 1a), which can be abstracted into a model that describes the dynamics and interactions of the system at that scale (Figure 1b). The macro-scale would then be the coarse-grained and dimensionally-reduced aggregate behavior in the form of the membrane potential (Figure 1c), which can be manipulated via current injection, and can be modeled in some abstract way as a set of interactions based on membrane potential (Figure 1d).

Notably, there is an astronomically large number of possible dimension reductions (scales) at which to model or intervene on a biological system. How to find the macro-scales that are most informative for modeling the system? Notably, a macro-scale may provide a more informative causal model compared to explicitly modeling the entire set of underlying channels, even though it is dimensionally-reduced. This is because noise might be minimized through the partitioning and coarse-graining at the macro-scale. This minimization of noise, and subsequent increase in information, has been called *causal emergence* [47].

In the next sections, we explore how to proceed with identifying cases of causal emergence formally. In order to have tractable models with explicitly formalized higher scales, throughout we make use of biological systems modeled as networks using open-source data. Specifically, we use gene regulatory networks (GRNs). First, we overview how to measure the degree of information such networks, focusing on assessing their amount of intrinsic noise and degeneracy. This is done using information theory. Second, we show how to create a macro-scale model from a given micro-scale model via dimension reduction. This is done by grouping nodes in a network into “macro-nodes.” Third, we apply these formalisms to a small GRN that controls mammalian cardiac development in order to identify the most informative model of cardiac development, which we show involves a macro-scale. Fourth, we apply these formalisms to the largest component of the gene network of *Saccharomyces cerevisiae*. Finally, we argue why we expect informative macro-scale models to be common across the biological sciences and why the mechanisms of life itself often operates at macro-scales.

## Information in the models of biological networks

As discussed in the previous section, in order to find the most informative scale to model a biological network at, one first needs a measure of information. Only in this way can the informativeness of different scales of a network be compared. Here we describe a measure that captures how much information is contained in a network of interactions of cells, proteins, or genes. Specifically, recent work has quantified the amount of information in a network of such interactions [48] using a measure called the *effective information* (*EI*) of a network. This measure assesses the uncertainty in the connectivity between the nodes of a network. The *EI* can be measured for complex networks which can include feedback, self-loops, or any other directed or undirected network architecture. Note here that these formalisms apply only to weighted or unweighted networks with directed connections, and which can be cyclic or acyclic, a common type of model in the biological sciences. Yet even with this limitation, the latest in creation of causal networks from nonlinear timeseries [49] and other methods make these techniques widely applicable to most biological subfields.

**Figure 2.**
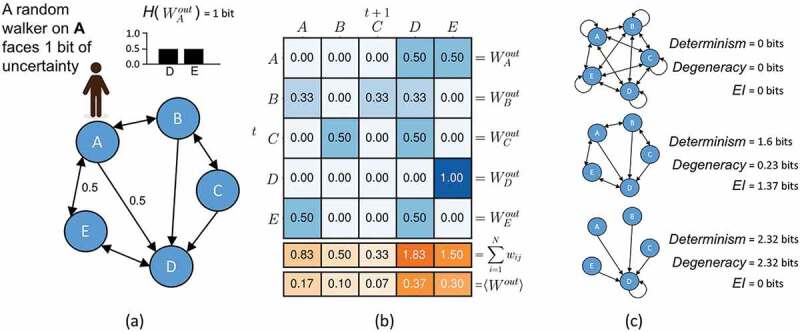
Measuring information in the causal structure of a network. (a) The entropy of a random walker’s next movement while standing on node *A* reflects the noise intrinsic to *A*’s outputs (note that this calculation requires normalizing the total weight of each node’s output to sum to 1.0). (b) <*W_i_^out^*> is the distribution of weight across the network (found by averaging across nodes in the network). (c) The *EI* is a function of the determinism minus the degeneracy of the network, which allows for the characterization of network architecture.

Specifically, for some network of *N* nodes, each node *v_i_* has an output, *W_i_^out^*, which is a vector of out-weights. This vector has an entropy, *H*(*W_i_^out^*), which reflects in bits the uncertainty [50] of a random walker standing on the node *v_i_* (as shown in Figure 2a). The average *H*(*W_i_^out^*), <*H*(*W_i_^out^*)>, is the amount of information lost due to the uncertainty of outputs in the network, i.e. the noise (indeterminism).

How this uncertainty is distributed also influences the amount of information contained in a network’s causal structure. This can be captured by examining the average *W^out^* (calculated in Figure 2b). The entropy of this vector, *H*(<*W_i_^out^*>), reflects how the total weight of the network is distributed. In a network where weight is distributed equally, *H*(<*W_i_^out^*>) is maximal at *log_2_*(*N*). In cases of complete degeneracy, which is when all nodes in the network connect solely to a single node, then *H*(<*W_i_^out^*>) = 0.0. Given these two quantities, the *effective information (EI)* of a network is:

$$Effective\;Information\;\left(EI\right)\;=\;H(<{W_{i}}^{out}>)\;-<H\left({W_{i}}^{out}\right)>$$

In cases of (all-to-all connectivity) where random walkers (reflecting the interactions or dynamics of the network) are moving in a completely unpredictable way, this would mean that *EI* will be 0.0, as well as in cases of complete degeneracy (see Figure 2c). Only in cases where each node of the network has a unique target, and therefore the dynamics are deterministic and non-degenerate, will *EI* be maximal. In this sense the *EI* of a system represents a quantification of *deep understanding*, defined in [12], as “knowing not merely how things behaved yesterday but also how things will behave under new hypothetical circumstances.” That is, in systems with high *EI* counterfactuals (hypothetical queries about one state instead of another) and interventions (such as the experimenter setting the system into a particular state) are more informative in that they contain more information about the future and past behavior of the system. Since it is only when *EI* is maximal that every difference in the system leads to a further unique difference, it also quantifies Gregory Bateson’s definition of information as “a difference that makes a difference” [51].

Bounded between 0 and *log_2_*(*N*), *EI*’s two fundamental components are:

$$Degeneracy\;=\;log_{2}\left(N\right)\;-H(<{W_{i}}^{out}>)$$

$$Determinism\;=\;log_{2}\left(N\right)\;-<H\left({W_{i}}^{out}\right)>$$

Degeneracy is uncertainty about the past, while indeterminism is uncertainty about the future. Together, the determinism and degeneracy fix the *EI* of the system, such that *EI* = *Determinism – Degeneracy* [48]. The determinism of the network is based on the average uncertainty a random walker faces at each node, measured by the entropy of each *W_i_^out^*. The degeneracy is based on the entropy of distribution of weights in the network and reflects how much overlap in targeting there is in the network (above and beyond the overlap due to indeterminism). How changes to network architecture control these properties are shown in Figure 1c, as well as how the two jointly make up the *EI*. Additionally, it should be noted that *EI* can also be expressed as the mutual information following a maximum entropy perturbation [52], thus it has a close connection to the control of an experimenter (for instance, the amount of information following the randomization of a variable in an experiment).

## How to find informative biological macro-scales

The most crucial difference between a macro-scale and its micro-scale is the amount of noise in the interactions of the system. This difference is captured by the differing *EI* values at the micro-scale vs. macro-scale. Our goal is to identify a scale with the maximum *EI*, which optimizes understanding and control by conceptually grouping some of the elements in a model into a “macro node”. That is, sometimes nodes in a network can be grouped in such a way that reduces the overall noise in the network, either by minimizing the degeneracy or maximizing the determinism [48]. Here we first overview the general formalisms for how to group nodes in a network, and then apply these techniques to a GRN derived from real data as an example of how to find informative biological macro-scales.

**Figure 3.**
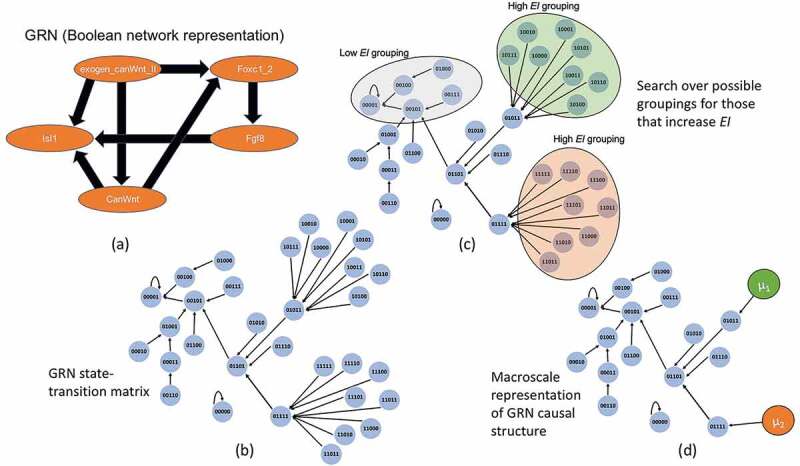
GRN coarse-graining. (a) The GRN as a Boolean network. Note *Isl1* has further projections into the larger cardiac regulatory network, but since these are not represented, it is instead given a self-loop as is traditional in Boolean analysis for nodes without outputs. (b) The expanded state-space wherein each node is a binary string of *exogen_canWnt_II, Foxc1_2, Fgf8, CanWnt, Isl1*. (c) Using a greedy algorithm that groups different sets of nodes together the possible partitions can be explored in a search for only those groupings that improve the *EI*. (d) Once the appropriate groupings are identified the network can be represented in its dimensionally-reduced format. Here the macro-nodes μ_1_ and μ_2_ are constructed in the simplest way possible, as a coarse-grain.

**Figure 4.**
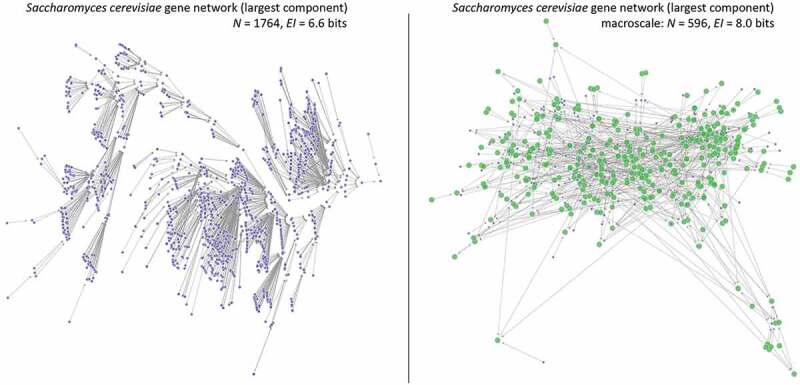
Macro-scale of Saccharomyces cerevisiae GRN. (Left) The largest component of the *Saccharomyces cerevisiae* gene regulatory state-space, derived from the Boolean network GRN representation. (Right) The same network but grouped into a macro-scale, found via the greedy algorithm outlined in [48]. There is a ~ 66% reduction in total states and an increase in the network’s *EI*. Green nodes represent macro-nodes in the new network.

The identification of a macro-scale entails the replacement of some set of nodes in the network, a subgraph *S*, with some single node that acts as a summary statistic for that subgraph’s behavior. This individual node is referred to as a *macro-node*, μ. Each node within the subgraph has some *W_i_^out^*, a vector that defines its outputs. In order for μ to appropriately capture the subgraph’s behavior it must be constructed from the set of each *W_i,S_^out^*. Note that in general if macro-scales are constructed correctly random walkers should behave identically on both the micro- and macro-scale [48], or within some degree of approximation, meaning the micro-scale dynamics are preserved at the macro-scale. Different macro-scales can preserve dynamics in different ways, meaning that the choice will always be system-dependent. For instance, macro-scales can be constructed as coarse-grains directly in the sense of averages, or as a more complicated weighted-average, but all macro-scales are dimension reductions in that they contain fewer nodes at the macro-scale (a smaller state space). For the system in Figure 3, all that is necessary is the simplest possible type of macro-node, which is a coarse-grain where the output of μ, *W*_μ_*^out^* is the average of the set of each *W_i,S_^out^*. At the end of this process some individual nodes A, B, C, etc., which are some subgraph of the network, are replaced by a macro-node, μ. However, for the system in Figure 4 we make use of a kind of macro-node based off of the stationary dynamics of the network, which have previously been shown to minimize dynamical differences in networks with stationary dynamics [48].

The replacing of a subgraph with a macro-node always reduces the dimension of the network by reducing the number of nodes. Causal emergence is defined as when a network’s macro-scale (after grouping) has more *EI* than its micro-scale (before grouping). This gain in *EI* at the macro-scale represents the amount of informational benefit from moving up in scale, which is a direct consequence of how much the given dimension reduction has minimized the noise. Here we search across the set of possible groupings in order to maximally improve the *EI* of the system.

As an example of how to find macro-nodes that minimize noise and degeneracy, we demonstrate the technique in a gene regulatory network of early cardiac development in mice [53]. A subset of the model is shown in Figure 3a, focusing around Wnt signaling (canWnt). The beginning of Wnt activation determines the mesodermal and cardiac cell lineage. Our subsection also includes regulatory factors Isl1 and Fgf8, which are critical for the heart looping stage of cardiogenesis, and are expressed within the pharyngeal endoderm. The exogenous signal canWnt II traditionally re-activates canWnt signaling at the cardiac crescent state.

Since gene regulation is often assumed to be essentially ON/OFF, it is common for GRNs to be represented as Boolean networks, as in Figure 3a. In order to examine the causal structure the Boolean network is expanded to its full state-space (Figure 3b). This expansion of the state-space creates a network of possible state-transitions, wherein the transition probabilities from each state are equivalent to a random walker on that node in the network, meaning that the uncertainty a random walker faces on a particular node is equivalent to the noise in the probability of change in gene expressions. At the micro-scale this state-space of the GRN has 2.78 bits of *EI*. A search is then conducted via an algorithm, the choice of which may vary depending on the architecture of the system. Note that one could use an array of different kinds of clustering or partitioning to identify viable candidates for macro-nodes, from dimensionality techniques like uniform manifold approximation method [54] to t-SNE [55]. So far a number of algorithms have already been compared for the purpose of finding macro-nodes in networks, such as one based on gradient descent, another on a greedy algorithm, and a third on spectral analysis [56]. Here, the spectral analysis algorithm is used, since it was deemed superior in terms of computational time and found the greatest increases of *EI* at the macro-scale compared to other algorithms so far. The choice of an algorithm is necessary since the space of possible macro-scales (all dimension reductions) is astronomically large.

In general, any algorithm seeking to identify causal emergence must look for groupings which increase the *EI* (since random groupings are highly likely to be poor candidates for macro-nodes). Only the macro-nodes that do improved the *EI*, as in Figure 3c, are kept in the macro-scale representation of the network. In this case the size of the state-space reduces from 32 states to only 18 states, and the *EI* increases to 2.9 bits, showing that over 40% of the network participates in the macro-scale and forms macro-nodes. Since the GRN state-space is deterministic, all of this informational gain comes from decreasing the degeneracy (from 2.21 bits of degeneracy at the micro-scale to 1.26 bits of degeneracy at the macro-scale), indicating that initial states can be predicted (or more accurately, retrodicted) easier from output or steady states.

What does this macro-scale representation tell an experimentalist or modeler? First, it provides a dimensionally-reduced and noise-minimized model of the causal structure. That is, the analysis replaces the state space of the micro-scale with a dimensionally-reduced state space of the macro-scale. This makes it easier to understand how the system temporally progresses in terms of its dynamics and “what causes what.” To see the advantages of this, consider a more traditional attractor analysis over the states of the system, such as examining which steady states follow an initial state. Usually, this is accomplished by running a model of the system forward in time given different initial states. Often, in a Boolean network all initial states lead to the same final resting or steady state. In this partial GRN all states eventually lead to the state {00001}, which is Isl1 being upregulated, except the state {00000}, which leads only to itself. Both states are the attractors of the system in that over its long-term dynamics it will always go to either one. Yet, in this attractor analysis information is lost in terms of what causes what, since only information about the two outputs is retained. While this tells the experimenter or modeler which end results to expect given an initial state, the order and nature of how those steady states are arrived at is left out of the analysis, and therefore their possible manipulations as well.

Additionally, what nodes get grouped into a macro-nodes tells us what interventional targets are meaningful within the system. Consider that of the two macro-nodes in the system {μ_1_, μ_2_}, each requires the activation of exogenous canWnt II. However, their set of underlying nodes are differentiated solely by the concurrent activation of Foxc1_2, which is not upregulated in μ_1_ and is upregulated in μ_2_. This tells us that it is solely Foxc1_2, rather than any other element in the network, that determines which causal path the network takes as long as exogenous canWnt II is activated. The macro-nodes capture which differences are actually relevant to the intrinsic workings of the system itself.

Macro-nodes always have either more deterministic or less degenerate connectivity, or both. They may also possess different properties than their underlying micro-nodes, such as memory or path-dependency [48]. Notably, since dimension reduction in general has no guarantee of increasing the determinism or minimizing the degeneracy, this indicates that modelers and experimentalists should in general be biased toward reduction, which fits with the historical success of reductionist approaches in science. However, in some circumstances reduction (fine-graining) may actually lose information by increasing noise or degeneracy. Measuring *EI* directly enables a principled way to assess this on a case-by-case basis in systems that can be modeled with networks.

While herein we focus solely on GRNs or protein interactomes, i.e. things that can be described as discrete Boolean networks with finite state spaces, the techniques we discuss are not limited to these sorts of biological systems. It should be noted that there are a number of existent algorithms or methods to dimensionally-reduce biological data from other sources. These include methods like quasi-steady state reduction (QSSR) for modeling biochemical pathways [57], such as enzymatic reactions [58]. Since continuous versions of *EI* exist [59], such techniques can be used to identify macroscales and then causal emergence in systems beyond just GRNs or other discrete models, although we do not consider them here.

## Why do biological networks have macro-scales?

We expect causal emergence to be common in biology, and at times higher levels in biology may represent significant dimension reductions. To see these ideas in action, consider the largest component of the gene network of *Saccharomyces cerevisiae* (shown in Figure 4), which is taken from the Cell Collective Database [60]. While a significantly larger directed network than the previously-analyzed cardiac development, it is still amenable to these techniques. Notably, in the search across different coarse-grainings of the state-space of the gene network of this common model organism undergoes a major dimension reduction when nodes are grouped to maximize *EI*, from 1764 nodes (each representing a state of the GRN) to merely 596. That is, the majority of the nodes in the network actually form macro-nodes. It is necessary to note here that random networks show no or extremely minimal causal emergence [48], that is, in purely randomly grown networks the vast majority of nodes do not form macro-nodes. It should also be noted that if the given GRN or interactome is incomplete or has incorrect connectivity, this analysis will be warped by the noise in the model’s construction, leading to an overestimation of causal emergence. This can be resolved in several ways, such as estimating how much noise is intrinsic to the model vs stems from the methodology, and such estimations have been applied to causal emergence in protein interactomes [61].

So what might be the reasons and benefits for a biological network, such as the gene network of *Saccharomyces cerevisiae*, to have the majority of its nodes participate in an informative higher scale? Below we offer three such reasons why we expect these sort of informative macro-scales to be common in biology, and why evolved systems have strong theoretical benefits to be multi-scale in their operation.

First, biology often must work with components in a noisy environment. Due to things like Brownian motion or the open nature of living organisms, it may be impossible to ever have deterministic relationships at a micro-scale. In a sense evolution deals with a constant source of noise, noise similar to that defined in information theory wherein sending a signal always has some degree of error [50]. Therefore, in order to make sure that causes lead to reliable or deterministic effects, biology necessarily needs to operate at the level of macro-scales. Indeed, error-correction is known to be important for the development and functioning of entire organisms themselves [62].

Second, emergent macro-scales have a high robustness due to their resistance to underlying component failure. The removal of a micro-node in a network will generally not affect it the same way as a macro-node. For example, a specific inhibitor that targets some particular micro-node in a biological causal structure, like an individual gene in Figure 3, would have minimal effect if its target was merely one part of a larger macro-node. This fits with evidence that evolution actively selects for robustness to node failures in biological causal structures like protein interactomes [14]. In such systems the impact of component failure is reduced due to the innate error-correction of macro-scale causation. Moreover, for a multiscale system to exhibit functional plasticity in changing circumstances (e.g. cells building to a specific morphological endpoint from diverse starting conditions in regulative development, body-wide remodeling, or regeneration [63,64]), its architecture must be such as to enable efficient modular control over its own space of possible actions.

Third, natural selection requires variability within a population. This has led to the proposal that the degeneracy observed in biological systems is critical for evolution to proceed [65]. This is because degeneracy provides a pool of variability for evolution to act on. However, at the same time, organisms need to be predictably consistent in their phenotypes, behavior, and structure to survive. As discussed, they need to have deterministic outcomes from noisy inputs. Therefore, having functions operate at macro-scales, whether those macro-scales arise from degeneracy or indeterminism, allows for both deterministic operation while preserving a pool of variability at the micro-scale. This may preserve population diversity while maintaining certainty over outcomes.

## Concluding perspectives

We have shown how information theory offers tools for objective analysis of the value of macro-scale models of biological systems, focusing on biological networks such as GRNs. Specifically, we made use of the effective information (*EI*) to assess the informativeness of a causal model, and then showed how EI can increase at macro-scales in both a GRN that plays an important role in mammalian cardiac development and also the largest component of the GRN of *Saccharomyces cerevisiae*.

While the formal tools outlined herein have undergone significant development since their first proposal [47,52] their applications in biological systems are just beginning. Although some have been skeptical about the use of information-based concepts in biology and whether it plays a key role in biological organization [66], recent advances have definitively shown how these can be rigorously applied; and indeed, software is now available to assist developmental biologists in calculating important information-theory metrics of genetic, physiological, and other data [67,68].

The techniques we’ve demonstrated apply not only to gene-regulatory and physiological circuits in development and regeneration [44,69], but also to important phenomena such as cancer [70]. However, note that the origin of data must be taken into account, as well as what a model network represents, in order for the analysis advanced here to be appropriately interpreted. In the case of inaccurate data, for instance, noise in the collection process may increase the apparent noise in the networks themselves, leading to an overestimation of causal emergence. Yet ultimately which subgraphs are good candidates for macro-nodes is a local phenomenon to those subgraphs, and *EI* scales with the growth of a network in a very patterned way [48]. So even in cases where the model used is incomplete or parts of it unknown, unless the unknowns directly change the connectivity of candidate subgraphs, which subgraphs are good candidates or group into macro-nodes with high *EI* should not be affected. However, in general, we recommend these procedures for well-studied biological networks or for large datasets wherein noise is averaged out. Applying these formalisms in continuous systems beyond discrete networks is a future area of research.

Given the advantages that multi-scale structures possess, which include error-correction, increased robustness, plasticity, and evolvability/learnability, we expect causal emergence to be common across nature. The increased informativeness of such higher scales are due to biology’s near-universal indeterminism and degeneracy and the ability of higher-level relationships to create certainty out of this uncertainty. If we are correct there should therefore be cross-species evidence that evolution selects specifically for multi-scale structure due to these advantages. This can be investigated by observing the evolutionary history of gene regulatory networks or protein interactomes, which is a significant direction for future research [61].

A further critical step will be the identification of high-information intervention targets in model systems and organisms. This is likely to first occur in simulations, including multi-scale models of development [71,72], regeneration [73–75], cancer [76,77], and physiology [78–80]. Ideally, this can provide proof that macro-scale interventions can not only control systems, but actually lead to more reliable downstream effects than their micro-scale alternatives, and therefore that biologists should adapt their scale of modeling and intervention to the system under investigation rather than taking a one-size-fits-all approach.

Many types of models in biology, from protein networks to physiological ones, can now benefit from a quantitative analysis of their causal structure, revealing the “drivers” of specific system-wide states and thus suggesting new strategies for rational interventions. Moving beyond traditional definitions of information [81] to analyzes of causation in networks across scales [82, Mayner, 83] can help drive new experimental work and applications in regenerative medicine, developmental biology, evolutionary cell biology, neuroscience, and synthetic bioengineering.
